# Drug-resistance patterns of *Mycobacterium tuberculosis* strains and associated risk factors among multi drug-resistant tuberculosis suspected patients from Ethiopia

**DOI:** 10.1371/journal.pone.0197737

**Published:** 2018-06-04

**Authors:** Eyob Abera Mesfin, Dereje Beyene, Abreham Tesfaye, Addisu Admasu, Desalegn Addise, Miskir Amare, Biniyam Dagne, Zelalem Yaregal, Ephrem Tesfaye, Belay Tessema

**Affiliations:** 1 Addis Ababa University, Department of Microbial, Cellular and Molecular Biology, Addis Ababa, Ethiopia; 2 Ethiopian Public Health Institute, Addis Ababa, Ethiopia; 3 Addis Ababa City Administration Health Bureau Health Research and Laboratory Services, Addis Ababa, Ethiopia; 4 St. Peter Hospital, Addis Ababa, Ethiopia; 5 Department of Medical Microbiology, College of Medicine and Health Sciences, University of Gondar, Gondar, Ethiopia; Indian Institute of Technology Delhi, INDIA

## Abstract

**Background:**

Multidrug drug-resistant tuberculosis (MDR-TB) is a major health problem and seriously threatens TB control and prevention efforts globally. Ethiopia is among the 30th highest TB burden countries for MDR-TB with 14% prevalence among previously treated cases. The focus of this study was on determining drug resistance patterns of *Mycobacterium tuberculosis* among MDR-TB suspected cases and associated risk factors.

**Methods:**

A cross-sectional study was conducted in Addis Ababa from June 2015 to December 2016. Sputum samples and socio-demographic data were collected from 358 MDR-TB suspected cases. Samples were analyzed using Ziehl-Neelsen technique, GeneXpert MTB/RIF assay, and culture using Lowenstein-Jensen and Mycobacterial growth indicator tube. Data were analyzed using SPSS version 23.

**Results:**

A total of 226 the study participants were culture positive for *Mycobacterium tuberculosis*, among them, 133 (58.8%) participants were males. Moreover, 162 (71.7%) had been previously treated for tuberculosis, while 128 (56.6%) were TB/HIV co-infected. A majority [122 (54%)] of the isolates were resistant to any first-line anti-TB drugs. Among the resistant isolates, 110 (48.7%) were determined to be resistant to isoniazid, 94 (41.6%) to streptomycin, 89 (39.4%) to rifampicin, 72 (31.9%) to ethambutol, and 70 (30.9%) to pyrazinamide. The prevalence of MDR-TB was 89 (39.4%), of which 52/89 (58.4%) isolates were resistance to all five first-line drugs. Risk factors such as TB/HIV co-infection (AOR = 5.59, p = 0.00), cigarette smoking (AOR = 3.52, p = 0.045), alcohol drinking (AOR = 5.14, p = 0.001) hospital admission (AOR = 3.49, p = 0.005) and visiting (AOR = 3.34, p = 0.044) were significantly associated with MDR-TB.

**Conclusions:**

The prevalence of MDR-TB in the study population was of a significantly high level among previously treated patients and age group of 25–34. TB/HIV coinfection, smoking of cigarette, alcohol drinking, hospital admission and health facility visiting were identified as risk factors for developing MDR-TB. Therefore, effective strategies should be designed considering the identified risk factors for control of MDR-TB.

## 1. Background

Tuberculosis (TB) continues to represent as a global major health challenge to the reduction of morbidity and mortality among millions of people every year. The health of approximately 10.4 million individuals worldwide are impacted annually by TB resulting in approximately 1.8 million TB-related deaths, with the majority (95%) of deaths were reported from resource-limited countries [1]. In Sub-Saharan countries, the prevalence of MDR-TB is high especially among previously treated TB cases when contrasted with new cases of TB [2]. Research has revealed that approximately 500,000 cases of MDR-TB emerge annually every year [3] and that approximately 3% of these cases receive treatment and that more than 100,000 deaths occur annually because of MDR-TB. In addition, as many as 10% of MDR-TB cases were extensively drug-resistant (XDR) [4]. MDR-TB is defined as resistance to both rifampicin and isoniazid; XDR is defined as MDR-TB with additional resistance to any fluoroquinolone and at least one of the three second-line injectable drugs: amikacin, capreomycin and kanamycin [5].

According to a recent World Health Organization (WHO) report of high TB-related burden countries, Ethiopia was identified as being among the thirty highest TB-burdened nations (TB, TB/HIV and MDR-TB) with TB remaining one of the Ethiopia’s leading causes of mortality. According to the 2017 WHO report, the prevalence of MDR-TB in Ethiopia was reported to be 2.7% and 14% among new and previously treated cases respectively with the prevalence of TB/HIV co-infection assessed as being 8% of the affected population [6]. Moreover, several studies done in Ethiopia showed that the prevalence of MDR-TB was 31.4% in Jimma [7], 28% in Addis Ababa [8], 46.3% in Addis Ababa [9] and 5% in Northwest Ethiopia [10]. Rapid transmission of MDR-TB is a major public health problem globally especially for resource-limited countries and represents a major challenge for TB control program. In addition, high prevalence of TB, poor treatment, limited access to health care, and several other related factors have constrained the ability of the sub-Saharan region, including Ethiopia to effectively control MDR-TB [11]. Finally, the rapid transmission of XDR-TB has recently emerged as yet another challenge for TB control program [12].

Drug-resistant strains of *Mycobacterium tuberculosis* (MTB) arise from spontaneous chromosomal mutations at a predictable low frequency, but a study done by Gandh et al. revealed that selection pressure that is caused by inappropriate utilize of anti-TB drugs results in the emerging of resistant MTB [13]. Similarly, a study done in Ethiopia identified long treatment, poor treatment follow up & interruption of treatment were identified as risk factors for significant increases in MDR-TB [14]. Other studies done in Ethiopia and China also revealed that HIV infection, cigarette smoking, alcohol drinking, overpopulated, and weak DOTS (Directly Observed Treatment Short-course) program were the major risk factors for spread of MDR-TB infection [7–9, 14, 15].

The global pattern of MDR-TB is not well known and little information is available regarding MDR-TB strains in a high TB/HIV prevalence countries like Ethiopia. MDR-TB is a result of unsuccessful TB control programs characterized by inappropriate TB treatment, and poor diagnostic capacity. In resource-limited countries such as Ethiopia, MDR-TB is public health threat due to poor adherence to treatment, delay of treatment and shortage of diagnostic center for MDR-TB [11]. In Ethiopia, an MDR-TB suspected case is defined as a patient who is a case of treatment failure; a symptomatic patient who had a close contact with confirmed MDR-TB patient; a patient from known high-risk group such as health workers; a patient who remains smear positive after 2 months of treatment (new cases); or remains smear positive after 3 months of retreatment with first-line treatment (retreatment cases such as defaulter, relapse) [16]. In all such cases, the development of enhanced diagnosis and treatment strategies are essential for controlling transmission of TB especially MDR-TB. Accordingly, this study focused on the identification of drug-resistance patterns of *Mycobacterium tuberculosis* strains among MDR-TB suspected patients and the associated risk factors for the development of MDR-TB in the study area.

## Materials and methods

### Study setting and design

A cross-sectional institution based survey was conducted between June 2015 and December 2016 in health facilities found in Addis Ababa, the capital city of Ethiopia. The study was conducted in selected health facilities. All samples were collected from study participants visiting the health facilities in Addis Ababa during the study period. Health facilities which provided laboratory services for MDR-TB diagnosis were selected from Addis Ababa city. Sample analysis was performed at Ethiopia Public Health Institute (EPHI) National TB Reference Laboratory, Ethiopia. The sample size was calculated using single population proportion formula considering the assumptions that at 95% confidence level with 5% precision and z value of 1.96 [17], and the 2014 Ethiopian national TB drug resistance survey report showed that the prevalence of drug-resistant TB among previously treated cases was 17.8% [18].

Thus, considering 10% nonresponse rate, the minimum sample size was 248 MDR-TB suspected cases. MDR-TB suspected cases are patients who are a case of treatment failure; a symptomatic patients who had a close contact with confirmed MDR-TB patient; patients from known high-risk group such as health workers; patients who remain smear positive after 2 months of treatment (new cases); or remain smear positive after 3 months of retreatment with first-line treatment [16].

In Ethiopia drug-resistant TB diagnosis has been carried out using the GeneXpert MTB/RIF assay and phenotypic drug susceptibility testing (DST). However, as GeneXpert MTB/RIF assays and DST are performed in only a few health facilities, MDR-TB suspected cases are referred to GeneXpert MT/RIF diagnostic sites. Since all MDR-TB suspected cases are referred to GeneXpert MT/RIF diagnostic sites, GeneXpert MT/RIF diagnostic sites found in Addis Ababa City were selected as study sites. Therefore, Addis Ababa Health research and Laboratory services (Addis Ababa Regional referral Laboratory), Teklehiamnot health center, and Saint Peter hospital were the study sites to recruit patients for enrollment in this study. Volunteer MDR-TB suspected patients who visited the health facilities during a study period were included as study participants. MDR-TB suspected patients who were seriously ill or unconscious, patients who were below the age of 12 years old, and patients who were not willing to participate in the study were excluded from the study.

### Sputum sample collection and laboratory analysis

Sputum samples from patients with pulmonary tuberculosis were collected into a sterile wide mouth 50 ml falcon tube a volume of 5 to 10 ml, and all specimens were stored at 2–8 ^o^C at sample collection sites until transported to EPHI TB laboratory using cold chain. Samples were analyzed using Ziehl-Neelsen Methods [19] and GeneXpert MTB/RIF assay [20] as the methods described. Moreover samples were cultured using LJ [21] and MGIT [22] methods for better yield; briefly, the samples were decontaminated with 4% NaOH-NALC and neutralized with phosphate-buffered saline (PBS), and then inoculated on LJ slants at 37°C for 8 weeks maximum [21] and in BACTEC™ MGIT 960 tubes (BD Diagnostics, Sparks, MD, USA) at 37°C for 42 days maximum [22]. All positive culture results were confirmed by using MPT64 antigen detection methods (Capilia TB) [23]. Phenotypic drug susceptibility test for rifampicin (RIF), isoniazid (INH), streptomycin (STR), ethambutol (EMB) and pyrazinamide (PZA) were performed with the Bactec MGIT 960 method. Briefly, 0.1 ml of a bacterial suspension with a McFarland standard was inoculated into a vial supplemented with reconstitution solution, and the concentration of drugs was 1.0 μg/ml for RIF, 0.1 μg/ml for INH, 5.0 μg/ml for EMB 1.0 μg/ml for STR and 100 μg/ml for PZA [22]. *Mycobacterium tuberculosis* strain H37Rv was used as a sensitive control for the susceptibility testing.

### Data analysis

Data were entered using Epinfo version 3.1 and exported to SPSS version 23 for analysis. Data completeness and consistency were checked by running frequencies of each variable. Bivariate analyses were carried out for categorical variables, and odds ratios were used to quantify the strength of association between potential risk factors and MDR-TB. Multiple logistic regressions were used to control the confounding effect of different variables while assessing the effect of each variable on the likelihood of MDR-TB occurrence. A p-value of 0.05 was used as the cut-off point for statistical significance. Variables having a p-value of at most 0.05 in bivariate analysis were included in the multivariate logistic regression model analysis.

### Ethical considerations

Ethical approval was obtained from Research and Ethical Review Committee of the Addis Ababa University and Ethiopian Public Health Institute. Written and or oral informed consent was taken from each study participant, and parent/guardian for those who were under age of 18 years old Permission was also obtained from study sites. Data and samples were collected and analyzed using codes so that the confidentiality of the patients & test result were maintained throughout the study period.

## Results

### Socio-demographic characteristics of the study participants

**Fig 1:**
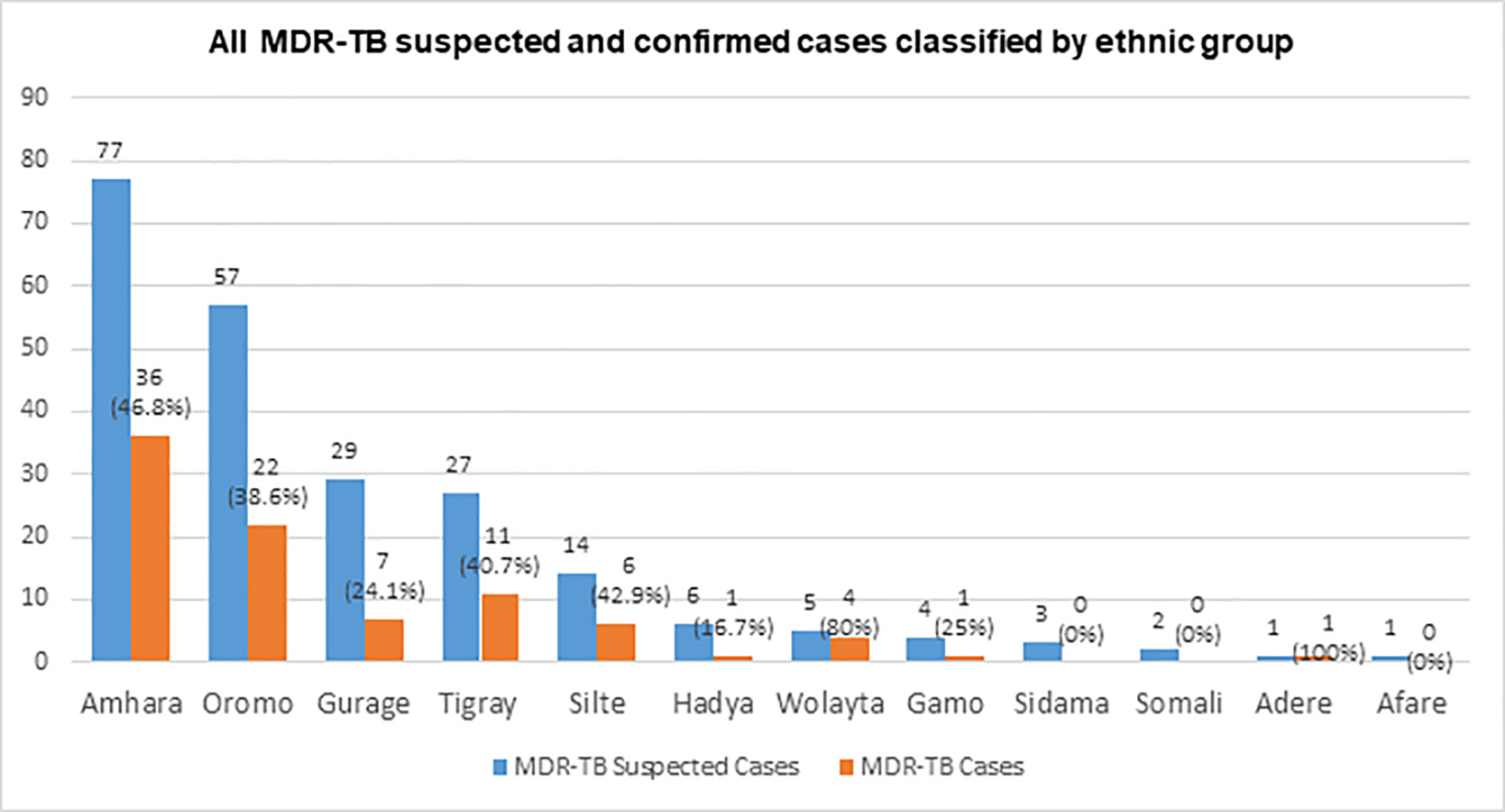
The proportion of MDR-TB among suspect cases classified by ethnic groups.

A total of 358 MDR-TB suspected cases were enrolled in this study, of which 226 (63.1%) were culture positive for MTB and 5 (1.4%) cases were positive for non-tuberculosis mycobacteria (NTM). Among MTB culture positive cases, majority 133 (58.8%) of cases were males, and 101 (44.7%) of the respondents were in the age group of 24–34 years with an average age of 34.4 years. Majority 213 (94.2%) of the respondents were living in an urban environment. Married individuals accounted for the majority 162(60.2%) the cases but 80(35.4%) were never married at all. Most 152 (67.2%) of the respondents were Orthodox by religion and majority 134 (53.1%) of the cases were from Amhara and Oromo ethnic group. Seventy two (31.8%) of the respondents attended high schools, and they were employed at private organization and 96 (42.5%) of participants had less than 1000 Birr (50 USD) income per month. More than half, 117 (51.7%) of the participants had 2 rooms in their residence. A majority [174 (77%)] of the participants lived in families with more than two family members. (Table 1 and Fig 1).

**Table 1:** Socio-demographic characteristics of MDR-TB suspected cases and MDR-TB confirmed cases, Addis Ababa, January, 2017(n = 226).

Variable	All MDR-TB suspected cases Number (%) (n = 226)	MDR-TB confirmed cases Number (%) (n = 89)
**Sex**		
Male	133 (58.8)	37 (41.6)
Female	93 (41.2)	52 (58.4)
**Age Group**		
15–24	27 (11.9)	4 (4.5)
25–34	101 (44.7)	53 (59.6)
35–44	68 (30.1)	23 (25.8)
45–54	20 (8.8)	6 (6.7)
Above 54	10 (4.4)	3 (3.4)
**Marital Status**		
Married	136 (60.2)	54 (60.7)
Single	80 (35.4)	31 (34.8)
Divorced	7 (3)	2 (2.2)
Widow	3 (1.3)	2 (2.2)
**Living Region**		
AA	208 (92.0)	83 (93.3)
Amhara	1 (0.4)	0 (0)
Dire Dawa	2 (0.9)	2 (2.2)
Oromia	12 (5.4)	4 (4.5)
SPNN	3 (1.3)	0 (0)
**Residence**		
Rural	13 (5.8)	4 (4.5)
Urban	213 (94.2)	85 (95.5)
**Education status**		
College	29 (12.8)	15 (16.9)
High School	72 (31.8)	26 (29.2)
Elementary	57 (25.1)	18 (20.2)
R&W	54 (23.9)	22 (24.3)
Illiterate	14 (6.2)	8 (9.0)
**Religion**		
Muslim	46 (20.4)	19 (21.3)
Orthodox	152 (67.3)	61 (68.5)
Protestant	28 (12.4)	9 (10.1)
Monthly Income in ETB		
No Income	3 (1.3)	6 (6.7)
100–1000	47 (20.8)	15 (16.9)
1001–2000	101 (44.7)	32 (36.0)
2001–3000	42 (18.6)	23 (25.8)
3001–4000	26 (11.5)	13 (14.6)
4001–5000	7 (3.1)	0 (0)
Occupation	3 (1.3)	6 (6.7)
Daily Laborer	28 (12.4)	9 (10.1)
Government Worker	30 (13.3)	14 (15.7)
House wife	26 (11.5)	13 (14.6)
Private Worker	72 (31.9)	23 (25.8)
Self-employed	67 (29.6)	29 (32.6)
Unemployed	3 (1.3)	1 (1.1)
**Number of rooms in residence**		
1–2	173 (76.5)	64 (71.9)
3–4	52 (23)	24(27)
5–6	1 (0.5)	1 (1.1)

ETB: Ethiopian Birr

### TB and treatment related conditions among MDR-TB suspected case

Among the MDR-TB suspected cases 196 (86.7%) cases were AFB positive and 128 (56.6%) cases were TB/HIV co-infected. One hundred sixty two (71.7%) cases were previously treated cases that had a history of TB treatment for more than a month in addition to this, 77(34.1%) cases had a history of family member infected by TB. Among the previously treated cases (n = 162), 148 (91.4%) were relapse and the remaining 9 (5.6%), and 5(3.1%) were treatment failure and defaulter cases, respectively. In addition, 26 (16.0%) cases had discontinued anti-TB drug during treatment time, of these 16(61.5%) cases had discontinued anti-TB drugs for a month or more and most 24 (92.3%) of the patients discontinued one time during their treatment period. One hundred and ninety (84.1%) cases were visiting health facilities for other reasons and 51(26.8%) cases were admitted to hospital. Eight-five (37.6%) cases had an antibiotic treatment history for other diseases, of which 32 (37.7%) had interrupted antibiotic treatment for more than once. Moreover, 42(18.6%) participants were self-reported frequent consumers of alcohol and 27(11.9%) reported themselves as being frequent cigarette smokers (Table 2).

**Table 2:** TB disease and other related conditions among MDR-TB suspected and MDR-TB confirmed cases, Addis Ababa, January, 2017(n = 226).

Variable	All MDR-TB suspected cases Number (%)	MDR-TB confirmed cases Number (%)
Family member Previously TB infected		
No	149 (65.9)	66 (74.2)
Yes	77 (34.1)	23 (25.8)
Treatment history of TB infected families		
No	3 (3.9)	0 (0)
Yes	74 (96.1)	23 (100)
Previously TB Infected		
No	64 (28)	16 (18)
Yes	162 (71.7)	73 (82)
Treatment history of previously TB infected cases		
No	0 (0)	0 (0)
Yes	162 (100)	73 (100)
Treatment interruption previously TB treated cases		
No	136 (84.0)	63 (86.3)
Yes	26 (16.0)	10 (13.7)
Duration of treatment interruption in days		
7–21	7 (26.8)	2 (20)
30–45	16 (61.5)	7 (70)
150	1 (3.8)	0 (0)
Unknown	2 (7.7)	1 (10)
Frequency of drug interruption		
1	24 (92.2)	9 (90)
3	1 (3.9)	0 (0)
Unknown	1 (3.9)	1 (10)
DOT treatment		
No	1 (0.6)	1(1.4)
Yes	161 (99.4)	72(98.6)
TB treatment history		
New	64 (28.3)	16 (18)
Retreatment	162 (71.7)	73 (82)
TB history		
New	64 (28.3)	16 (18)
Defaulter	5 (2.2)	3 (3.4)
Relapse	148 (65.5)	63 (70.8)
Treatment failure	9 (4.0)	7 (7.9)
AFB Results		
Negative	30(13.3%)	2(2.2%)
Positive	196(86.7%)	87(97.8%)
HIV status		
Positive	128 (56.6)	71 (79.8)
Negative	98 (43.4)	18 (20.2)
Antibiotic treatment history (frequently)		
No	141 (62.4)	45 (50.6)
Yes	85 (37.6)	44 (49.4)
Antibiotic treatment interruption		
No	53 (62.3)	29 (65.9)
Yes	32 (37.7)	15 (34.1)
Alcohol drinking frequently		
No	184 (81.4)	61 (68.5)
Yes	42 (18.6)	28 (31.5)
Cigarettes smoking		
No	199 (88.1)	73 (82)
Yes	27 (11.9)	16 (18)
Health facility visiting		
Yes	190 (74.1)	82(92.1)
No	36 (15.9)	7 (7.9)
Hospital admission		
No	175 (77.4)	55 (61.8)
Yes	51 (22.6)	34 (38.2)

AFB: Acid fast Bacilli; DOT: Directly Observed Treatment; HIV: Human Immunodeficiency Virus

### *Mycobacterium tuberculosis* identification and drug susceptibility test results

Among 226 *Mycobacterium tuberculosis* isolates, the majority 123 (54.4%) of isolates were resistant to at least one of the five first-line anti TB drugs (RIF, INH, PZA, EMB, and STR), however, the remaining 104 (46.0%) isolates were susceptible to all first-line anti TB drugs. Moreover, resistance to INH, STR, RIF, EMB and PZA were 110 (48.7%), 94 (41.6%), 89 (39.4%), 72 (31.9%), and 70 (30.9%), respectively. Furthermore, the proportion of drug-resistance pattern among previously treated cases (n = 162) were 87 (53.7%), 75 (46.3%), 73 (45.1%), 59 (36.4%), and 59 (36.4%), and 57 (35.2%), INH, STR, RIF, EMB and PZA, respectively (Table 3).

**Table 3:** Drug resistance pattern in Mycobacterium tuberculosis complex isolates among retreatment and new MDR-TB suspected cases Addis Ababa, January, 2017(n = 226).

Drug Resistance Pattern	All case (n = 226) Number (%)	Previously treated cases (n = 162) Number (%)	New cases (n = 64) Number (%)
**Any Resistance**	**123 (54.4)**	**95 (58.6)**	**28 (43.8)**
EMB	72 (31.9),	59 (36.4)	13 (20.3)
INH	110 (48.7),	87 (53.7)	23 (35.9)
STR	94 (41.6)	75 (46.3)	19 (29.7)
PZA	70 (30.9)	57 (35.2)	13 (20.3)
RIF	89 (39.4)	73 (82)	16 (25.0)
**Mono Resistance**	**20 (8.8)**	**12 (7.4)**	**8 (12.5)**
EMB	4 (1.8)	4 (2.5)	0 (0.0)
INH	7 (3.1)	4 (2.5)	3 (4.7)
STR	7 (3.1)	3 (1.9)	4 (6.3)
PZA	2 (0.9)	1 (0.6)	1 (1.6)
**Multi drug Resistance(MDR)**	**89 (39.4)**	**73 (45.1)**	**16 (25.0)**
RIF + INH	5 (2.2)	5 (3.1)	0 (0)
RIF + INH + EMB	3 (1.3)	2 (1.2)	1 (1.6)
RIF + INH + STR	10 (4.4)	8 (4.9)	2 (3.1)
RIF + INH + PZA	2 (0.9)	1 (0.6)	1 (1.6)
RIF + INH + EMB + STR	6 (2.7)	6 (3.7)	0 (0)
RIF + INH + EMB + PZA	4 (1.8)	3 (1.9)	1 (1.6)
RIF + INH + STR + PZA	5 (2.2)	4(2.5)	1 (1.6)
RIF + INH + EMB + STR + PZA	52 (23.0)	43 (26.5)	9 (14.1)
Poly Resistance* **(Non MDR)**	**13 (5.8)**	**9 (5.6)**	**4 (6.3)**
EMB + INH	2 (0.9)	1 (0.6)	1 (1.6)
INH + STR	6 (2.7)	3 (1.9)	3 (4.7)
EMB + INH + STR	1 (0.4)	1 (0.6)	0 (0.0)
INH + STR + PZA	3 (1.3)	3 (1.9)	0 (0.0)
EMB + INH + STR + PZA	1 (0.4)	1 (0.6)	0 (0.0)

INH: Isoniazid, RIF: Rifampicin, STR: Streptomycin, EMB: Ethambutol, PZA: Pyrazinamide

*Poly Resistance is a drug resistance to two and more drugs without the combination of INH and RIF

### Patterns of multidrug-resistance

The prevalence of multi-drug-resistant TB was 89 (39.4%), of which 73 (82.0%), 65 (73.0%) and 63 (70.8%) were additionally resistant to STR, EMB and PZA anti-TB drugs, respectively. In addition, among multi-drug-resistant TB cases, 52/89 (58.4%) were resistance for all first-line anti TB drugs (RIF, INH, PZA, EMB, and STR). Moreover, the prevalence of Multi-drug-resistant TB among previously treated cases was 73/162 (45.1%) whereas 16/64 (25%) were among new cases. A higher multi-drug-resistance rate was observed among previously treated cases 73/89 (82.0%) compared with new cases, and a substantial drug-resistance pattern was observed on STR, PZA, and EMB drugs with 64 (87.7%), 53 (72.6%) and 50 (68.5%) isolates respectively. Out of 89 MDR-TB confirmed cases, more than three-fourth, [71 (79.8%)] were HIV positive patients, and majority 87 (97.8%) of the MDR-TB cases were AFB positive (Table 2). However, 3 (1.3%) isolates had discordant results from MGIT and GeneXpert methods; both isolates had RIF susceptible results from MGIT but they were found to be RIF resistant by GeneXpert assay. The other one isolate had a resistant result from MGIT and susceptible with GeneXpert assay. When we evaluated the performance of GeneXpert method against MGIT for DST, we found that sensitivity result was 99.1% with 99.6% specificity.

Seventy-three (82.0%) MDR-TB cases were previously treated cases and the remaining 16 (18.0%) were new cases. Moreover 23 (25.8%) MDR-TB cases had a history of family member infected by TB. Among the previously treated cases, the majority [63 (86.3%)] of the MDR-TB cases were relapse cases, and 7 (9.6%) cases had discontinued their use of an anti-TB drug during treatment and 5/7 (71.4%) the cases discontinued anti-TB drugs for more than a month. Moreover 82 (92.1%) MDR-TB cases were visiting health facilities for other reasons and 20 (22.5%) cases were admitted to hospital. More than a quarter, [23 (25.8%)] had an antibiotic treatment history for other diseases, while 8/23 (34.7%) of these cases had interrupted antibiotic treatment more than once. The majority of MDR-TB cases 52 (58.4%) were males and 85 (95.5%) MDR-TB cases were lived in an urban environment. Fifty-three (59.6%) of the MDR-TB cases were in the age group of 25–34 years and 54 (60.7%) the cases were married and more than half, [58 (65.2%)] of the cases were from Amhara and Oromo ethnic group (Tables 1 and 2, Fig 1).

### Risk factors associated with multidrug resistance development

In a univariate analysis of different variables with the development of MDR-TB, TB/HIV co-infection, previously TB infected cases, a family member who had previously TB infected cases, antibiotic taking, alcohol drinking, age group between 25 & 34 years, cigarette smoking, health facility visits and hospital admission appear to represent significant risk factors for MDR-TB (p<0.05). Moreover, multivariate analysis indicated that MDR-TB is significantly associated with hospital admission, (AOR = 3.49, p = 0.005) health facility visit, ((AOR = 3.34, p = 0.044), TB/HIV co-infection, (AOR = 5.59, p = 0.00) alcohol drinking (AOR = 5.14, p = 0.001) and cigarette smoking (AOR = 3.52, p = 0.045). Patients admitted to hospital and visited health facilities and those who were self-reported as being frequent cigarette smokers were three times more likely to develop MDR-TB when compared to those who did not fit these profiles. Moreover, TB/HIV co-infection and drinking alcohol were five times more likely to have MDR-TB when compared with those HIV negative cases and TB patients who did not drink alcohol (Table 4).

**Table 4:** Univariate and multivariate logistic regression result of risk factors for development of MDR-TB, Addis Ababa, January, 2017 (n = 226).

Variable	MDR-TB N (%)		Crude Odd ratio (95%)	P-Value	Adjusted Odd ratio (95%)	P-Value
	Yes	No				
Age Group						
15–24	4 (14.8)	23 (85.2)	1		1	
25–34	53 (52.5)	48 (47.5)	6.35 (2.05 19.70)		3.73 (0.51 27.37)	0.261
35–44	23 (33.8)	45 (66.2)	2.94 (0.91 9.51)	0.001	0.65 (0.13 3.27)	
45–54	6 (30.0)	14 (70.0)	2.46 (0.59 10.29)		0.78 (0.34 9.28)	
55≥	3 (30.0)	7 (70.0)	2.46 (0.44 13.75)		2.29 (0.34 15.36)	
Cigarettes smoking						
Yes	73 (36.7)	126 (63.3)	2.51 (1.11 5.70)	0.045	3.52 (1.03 12.05)*	0.045
No	16 (59.3)	11 (40.7)				
Alcohol drinking						
Yes	61 (33.2)	123 (66.8)	4.03 (1.98 8.12)	0.000	5.14 (1.98 13.33)*	0.001
No	28 (66.7)	14 (33.3)	1			
HIV Status						
Positive	71 (55.5)	57 (44.5)	5.54 (2.98 10.28)	0.000	5.59 (2.65 11.75)*	0.000
Negative	18 (18.4)	80 (81.6)	1			
Antibiotic treatment history						
Yes	45 (31.9)	96 (68.1)	2.29 (1.32 3.98)	0.000	1.83 (0.88 3.80)	0.106
No	44 (51.8)	41 (48.2)	1			
TB treatment history						
Retreatment	73 (45.1)	89 (54.9)	2.46 (1.91 4.69)	0.000	2.21 (0.80 5.89)	0.110
New	16 (25.0)	48 (75.0)	1			
TB History						
New	16 (25.0)	48(75.0)	1		1	
Defaulter	3 (60.0)	2 (40.0)	4.50 (0.69 29.38)	0.012	8.26 (0.86 78.95)	0.067
Relapse	63 (42.6)	85 (57.4)	2.22 (1.16 4.27)		3.56 (0.26 50.16)	
Treatment failure	7 (77.8)	2 (22.2)	10.5 (1.97 55.8)		2.78 (0.48 16.14)	
Previously TB infected family member						
Yes	66 (44.3)	83 (55.7)	0.54 (0.30 0.96)	0.037	0.67 (0.18 2.45)	0.541
No	23 (29.9)	54 (70.1)	1		1	
Health facility Visit						
Yes	7 (19.4)	29 (80.6)	3.15 (1.31 7.54)	0.019	3.34 (1.03 10.78)*	0.044
No	82 (43.2)	108 (56.8)	1			
Hospital admitted						
Yes	55 (31.4)	120 (68.6)	4.36 (2.25 8.48)	0.000	3.49 (1.45 8.40)*	0.005
No	34 (66.7)	17 (33.3)	1			

* The odds ratio indicated that there was significant association between dependent variable **(**MDR-TB) and independent variables; N: number

## Discussion

In this study, about 72% of cases were previously treated cases that had a history of TB treatment for more than a month and 34.1% cases had a history of a family member infected by TB. In addition 16.0% of the cases had discontinued anti-TB drug during the treatment period. More than 56% of the cases were TB/HIV co-infected patients, and about 80% of the TB/MDR-TB cases were HIV positive patients. The high prevalence in our study could be attributed to the fact that HIV-positive patients are more likely to develop TB/MDR-TB than HIV negative patients due to their immunocompromised status. In supporting our finding, several studies revealed that HIV infection was the major associated risk factor for spread of MDR-TB infection in population [7–9, 14, 15]. Moreover, the study also found that more than 95% of the MDR-TB cases lived in an urban environment. Evidence from a previous study showed that patients who live in an urban area are more likely to develop drug-resistant TB due to slums/overcrowded area that favor for transmission of TB/MDR-TB [7].

Furthermore, the highest rate of MDR-TB patients were also in the age group of 25–34 years, a finding that is consistent with other studies done in Ethiopia [7, 24, 25]. The highest rate of MDR-TB in this age group might possibly be due to this age cohort’s high mobility and high-risk behavior could possibly expose them to greater risk of acquiring TB as well as to a tendency to interrupt TB treatment. In another finding, this study identified that 54% of the study’s population was resistant to any first-line anti-TB drug, a finding that was lower than previous studies done in Addis Ababa (72.9%) [9] and Southwest, Ethiopia (58.6%) [7]. In accounting for this difference, it is possible that, as our study included both previously treated and new cases, a new TB patient is less likely to develop drug resistant TB.

The highest proportion of drug resistance was observed for INH (49%). This is comparable to the studies done in Southwest Ethiopia (51%) [7] and Addis Ababa (56.1%) [9]. However, as our finding on 49% INH resistance was slightly higher than previous studies done in Ethiopia such as 44% [8] and 42.7% [9]), the high proportion of isoniazid resistance has significant implications since it is an essential drug during the course of TB treatment and a prophylaxis for latent TB infected individuals, HIV/AIDS patients and household contacts of smear-positive pulmonary TB cases. In addition, our study found that, at a rate of 41.6%, streptomycin resistance was comparable with a study done in Southwest, Ethiopia (42.9%) [7], although appreciably higher than other Ethiopian studies such as 21% [9] and 28% [8]. The high resistance to streptomycin could be due to the common use of the drug for treatment of any bacterial infections, poor treatment practice and early introduction for treatment [26].

The proportion of drug resistance for rifampicin drug was rated third with 39.4% and all rifampicin drug-resistant cases were also resistant to Isoniazid which are MDR-TB cases. This finding concurs the present practice of TB programs to use RIF resistance as a surrogate marker for MDR-TB diagnosis and second line anti-TB drugs treatment initiation. Our finding was higher than the findings of previous studies done in Addis Ababa (33.3%) [8] and Southwest, Ethiopia (32.9%) [7]. The high prevalence in our finding might be that the study was conducted on population of MDR-TB suspected patients. Furthermore, the ethambutol drug resistance was ranked fourth with 31.9%. It is comparable with a study done in Southwest, Ethiopia that the proportion of any ethambutol resistance was about 29% [7]. Although it is the first-line drug, it is also included in the regimen of second-line drugs for MDR-TB treatment. Hence the high rate of ethambutol resistance could be a challenge for MDR-TB treatment in the future [27]. Furthermore, resistance to PZA was 30.9%, it is well known that PZA is a cornerstone anti TB drug because of its unique ability to eradicate persistent bacilli, that allowed treatment shortening from 9 months to 6 months [28] and it is continuing as an important drug for susceptible and MDR-TB treatment [29]

In terms of prevalence of MDR-TB, the prevalence of MDR-TB was 39.4%, our finding was somewhat higher than the previous study done in Southwest, Ethiopia 31.4% [7], and compared with Ethiopian national prevalence for previously treated cases, it was twofold higher (17.8%) [6]. In addition, the proportion of MDR-TB among previously treated was 45.1%. This finding is in agreement with a study done in Addis Ababa, Ethiopia (46.3%) [9] and India (47.1%) [30]. However, it was higher than other studies conducted in Ethiopia (28%) [8] and Northwest Ethiopia (13.9%). The plausible reasons for high prevalence in our finding might be that the study was conducted among population of MDR-TB suspected patients;, the nature of this population included in the studies, and there might also be geographical variation in the level of drug resistance. Our assumption was supported by Mekonnen and colleagues [31].

In another finding, the percentage of MDR-TB among previously treated cases was significantly higher (45.1%) compared to new TB cases (25%) It is well documented that previously treated cases are more likely to develop MDR–TB than new patients [32]. High rates of MDR-TB among previously treated cases can be influenced by the acquisition of resistance in the intensive and continuation phases of treatment or by the rate of primary MDR-TB infection [33]. In addition, the rate of MDR-TB among new cases was 25% was higher than Ethiopian national prevalence for new cases was 2.7% [6], and in other studies done in Ethiopia including those in Debre Markos (10.7%) [34], Northwest Ethiopia (2.3%) [31], and East Gojjam (1.29%) [32]. This finding might indicate a significant public health threat given that there would appear to be a progressive MDR-TB transmission in the population. So this finding could be a good indicator for a need to strengthen the health system towards a more effective TB treatment, diagnostic, and prevention and control Program.

Furthermore, while it is well known that the drug-resistant TB is a result of chromosomal alterations due to mutations or deletions, there are several factors related to TB control program that have a significant impact on the increasing and transmission of drug-resistant TB [35]. Our study revealed that MDR-TB infection had a statistically significant association with patients admitted to hospital (p<0.005), patients who visited health facilities (p<0.005), HIV positive patients (p<0.005), patients who were frequent cigarette smokers (p<0.005) and patients who frequently drink alcohol (p<0.005). All of these factors would appear to be predictors for MDR-TB. This finding is in agreement with studies done in Addis Ababa, Ethiopia [8, 9] and China [36]. Moreover, several pieces of evidence revealed HIV/AIDS, overcrowding and lack of compliance with DOTS program, are also the potential risk factors for the development of MDR-TB infection [7, 14, 15, 37]

In another part of this study, we tried to evaluate the performance of GeneXpert method against MGIT, which is a standard method, for DST, we found that 3 isolates had discordant results from MGIT and GeneXpert methods (99.1% sensitivity and 99.6% specificity), two isolates had RIF susceptible results from MGIT with GeneXpert resistance results. This situation might be due to the existence of *Mycobacterium tuberculosis* strains with borderline of susceptible [38]. Moreover, one isolate was found to be resistance from MGIT and susceptible with GeneXpert assay, It is well documented that about 5% RIF resistance isolates did not have any mutation in the rpoB gene, and the mechanism of resistance could be due to intrinsic drug resistance mechanism in which it is attributed to its unique cell wall properties, including the presence of mycolic acids, which constitute a very hydrophobic barrier responsible for resistance to certain antibiotics [39]. So this might be preliminary finding for further study on the performance of GeneXpert assay among MDR-TB suspected cases in Ethiopia.

In general, the findings presented in this paper would tend to confirm that patient progressive acquisition of drug resistance during TB treatment is a significant contributor to higher rates of MDR-TB since anti-TB drug treatment suppress the growth of susceptible TB isolates while, at the same time, favor the multiplication of the existing drug-resistant isolates as described by Mekonnen et al. [31]. MDR-TB control programs currently focus on factors implementing the guidelines for TB control programs such as early case detection, treatment adherence, infection prevention and administrative and logistic issues [40]. As this study provides information on patterns of drug-resistant TB and associated risk factors among previously treated and new cases, it is proposed that this study’s findings could be applied to an increased understanding of factors associated with the development of MDR-TB in the population and, hence, to ways in which to improve planning associated with ways by which to reduce MDR-TB.

## Conclusion

In conclusion, the present study has revealed that the prevalence of multidrug-resistant tuberculosis in the study area was higher compared to WHO data and previous studies done in Ethiopia and that the proportion of MDR-TB among previously treated patients and young age group was also higher than previous studies. The major risk factors for the development of MDR-TB were TB/HIV co-infection, frequent cigarette smoking, frequent consumption of alcohol, hospital admission, and a history of visits to health facilities. Finally, this study would conclude that, as a major public health threat is represented by the finding that there is a progressive MDR-TB transmission in the population especially in the productive age group of the population, actions should be taken to improve outreach to populations at risk of MDR-TB if Ethiopia is to avoid an environment in which MDR-TB continues to increase its impact on the health of the nation.

Therefore, TB patients suspected for MDR-TB should be identified in a timely manner and treated according to treatment guideline, and the country should focus its efforts on developing a strategy designed toward early detection and treatment of MDR-TB cases in the population, and monitoring systems to investigate the trend of MDR-TB incidence and efficacy of MDR-TB treatment regimens. Moreover, further studies should be supported to determine the transmission dynamics of multidrug-resistant strains using genotyping tools as well as studies devoted to increasing and refining the public health community’s understanding of risk factors for the development of MDR-TB in the population.

## Supporting information

S1 File All Socio-demographic and drug susceptibility test result data xls File. (XLSX)
